# Survival outcomes and surgical morbidity based on surgical approach to pulmonary metastasectomy in pediatric, adolescent and young adult patients with osteosarcoma

**DOI:** 10.1002/cam4.6491

**Published:** 2023-10-06

**Authors:** Christopher Kuo, Jemily Malvar, Yueh‐Yun Chi, Eugene S. Kim, Rachana Shah, Fariba Navid, James E. Stein, Leo Mascarenhas

**Affiliations:** ^1^ Department of Pediatrics, Division of Hematology‐Oncology, Cancer and Blood Disease Institute Children's Hospital Los Angeles Los Angeles California USA; ^2^ Keck School of Medicine University of Southern California Los Angeles California USA; ^3^ Department of Surgery, Division of Pediatric Surgery Children's Hospital Los Angeles Los Angeles California USA

**Keywords:** osteosarcoma, pulmonary metastasectomy, thoracoscopy, thoracotomy

## Abstract

**Background:**

Thoracotomy is considered the standard surgical approach for the management of pulmonary metastases in osteosarcoma (OST). Several studies have identified the advantages of a thoracoscopic approach, however, the clinical significance of thoracotomy compared to thoracoscopy is yet to be evaluated in a randomized trial.

**Aims:**

The primary aim was to determine the survival outcomes in OST patients based on surgical approach for pulmonary metastasectomy (PM) and secondary aim was to assess the post‐operative morbidities of OST PM through various surgical approaches.

**Materials and Methods:**

We conducted a single institution retrospective study to compare survival outcomes and surgical morbidity according to the surgical approach of the management of pulmonary metastases in patients with OST.

**Results:**

Sixty‐one patients with OST underwent PM. Twenty‐one patients were metastatic at diagnosis and underwent PM during primary treatment; nine had thoracotomy, six thoracoscopy, and six combined thoracoscopy with thoracotomy (CTT). Forty‐three patients with first pulmonary relapse or progression underwent PM; 18 had thoracotomy, 16 thoracoscopy and nine CTT. There was no difference in survival between surgical approaches. There were significantly more postoperative morbidities associated with thoracotomy for initial PM (pain and postoperative chest tube placement), and for PM at first relapse (pneumothoraces, pain, Foley catheter use and prolonged hospitalizations).

**Conclusion:**

Our study demonstrates that patients with OST pulmonary metastases have comparable poor outcomes despite varying surgical approaches for PM. There were significantly more postoperative morbidities associated with thoracotomy for PM. Surgical bias and other competing risks could not be assessed given the limitations of a retrospective study and may be addressed in a prospective trial evaluating surgical approach for PM in OST.

## 1 INTRODUCTION

Approximately 20% of patients with osteosarcoma (OST) present with pulmonary metastases at diagnosis and over a third of patients with localized disease will develop lung metastases.^1^, ^2^ The lung continues to be the most common site of relapse for patients with OST. In the European and American Osteosarcoma Study of more than 2000 patients with OST, approximately 92% of relapses involved the lungs.^3^ Despite intensive multimodal therapy, patients with pulmonary disease have dismal outcomes with long‐term survival consistently less than 40%.^4^ Surgical control of macroscopic disease both at primary and metastatic sites in OST is considered essential for cure. Although thoracoscopic approach may limit morbidity by decreasing length of stay and postoperative pain, there is a theoretical concern that the inability to manually palpate sub‐centimeter lesions not apparent on preoperative computed tomography (CT) scan (due to inherent limited resolution) may leave unresected disease behind which may in turn impact outcome.^5^ Thus in OST, open thoracotomy has been the standard approach for pulmonary metastasectomy (PM). Limited information is available on the impact of survival for each surgical approach due to the lack of prospective trials. The primary aim of this study was to determine the survival outcomes based on surgical approach in the management of pulmonary metastases in patients with OST. The secondary aim was to assess postoperative morbidities of OST PM through various surgical approaches.

## 2 MATERIALS AND METHODS

Medical records of all patients with OST and pulmonary metastases diagnosed and treated between January 1, 2004 and December 31, 2018, at Children's Hospital Los Angeles were reviewed. Only patients that underwent metastasectomy during primary treatment or at first relapse were analyzed. Data including age at diagnosis, sex, primary site, presence or absence of metastatic disease, sites of metastases, location, treatment details, surgical approach to pulmonary metastases, histologic response of primary tumor to chemotherapy, surgical morbidities (infection, pneumothorax/emphysema, pain, Foley catheter usage, chest tube placement, and days of hospitalization), date of last follow‐up and vital status were collected. Requirements for epidural and/or patient‐controlled analgesia (PCA) with or without continuous narcotic infusion were used as surrogates for pain. Good histologic response following chemotherapy was defined as <10% residual viable tumor, and poor histologic response as ≥10% residual viable tumor.^6^, ^7^, ^8^ Patients were subcategorized based upon the surgical approach: thoracotomy, thoracoscopy, and combined thoracoscopy with thoracotomy (CTT). We define CTT as the utilization of thoracoscopy as the initial approach with conversion to thoracotomy in the same surgical episode. Data were recorded in REDCap® database. The study was approved by the Institutional Review Board prior to data collection (CHLA‐19‐00107).

### 2.1 Statistical analysis

Event‐free survival (EFS) and overall survival (OS) were defined as the time from diagnosis to the occurrence of the qualifying event (relapse, second malignancy, or death for EFS and all‐cause mortality for OS). EFS and OS were also assessed from time of first relapse to occurrence of qualifying event since first relapse. Survival was evaluated using the Kaplan–Meier method,^9^ with standard errors estimated by the Greenwood formula.^9^ The log‐rank test was utilized to evaluate differences in EFS and OS between disease and surgical variables. Fisher's exact test and Kruskal–Wallis sum rank test were used to evaluate postoperative morbidities among the surgical groups, as appropriate. Unless otherwise stated, all analyses were performed using two‐sided tests; the significance level set at *p* < 0.05 and completed using the statistical software Stata (version 17).^10^

## 3 RESULTS

### 3.1 Patient characteristics

Sixty‐one patients with OST who underwent PM were identified through medical record review (Table 1). Majority of the patients were male (57%) and the median age at diagnosis was 13 years (range, 5–21). Over half of the patients were Hispanic and approximately 23% were white/non‐Hispanic. The median follow‐up time for patients that were alive at last follow‐up was 6.2 years from diagnosis (range 0.6–14.6 years; *n* = 46). The median follow‐up for the patients without first relapse or death was 7.1 years from diagnosis (range 1–10.5 years; *n* = 10). Majority of primary tumors were in the extremities (92%), specifically in the distal femur (39%) followed by the proximal tibia (30%). Lungs were the predominant metastatic site, while four patients (7%) also had bone metastases. Poor histologic response to neoadjuvant chemotherapy in the primary tumor was observed in over half the patients (54%), 58 (95%) received methotrexate (M), doxorubicin (A), and cisplatin (P), cisplatin was replaced with ifosfamide in two patients due to significant baseline hearing loss and no chemotherapy information was available for one patient. Of the 61 patients with OST who underwent PM, 37 (61%) were localized and 24 (39%) were metastatic at initial diagnosis. Of the 24 patients that were metastatic at diagnosis, 15 (63%) had bilateral lesions and 9 (38%) had unilateral lung lesions. Of the 61 patients, 11 (18%) did not relapse, 20 (33%) had one relapse, 14 (23%) had two relapses, 10 (16%) had three relapses, 4 (7%) had four relapses and 2 (3%) had five relapses. Of the 24 patients that were metastatic at diagnosis, nine were in first continuous remission and 15 had a relapse. Of the 15 patients that were metastatic at diagnosis and had at least one relapse, two were ipsilateral relapses, four had bilateral pulmonary metastases at diagnosis and relapsed unilaterally, seven had bilateral lung metastases at diagnosis and relapsed in bilateral lungs, one had right pulmonary metastases at diagnosis and relapsed bilaterally, and one had bilateral lung metastases at diagnosis with an extrapulmonary relapse. Of the 37 patients that were localized at diagnosis and underwent metastasectomy for first relapse, two had no pathologic evidence of OST and 35 had OST confirmed. Of those 35 patients with localized OST that had first relapse confirmed, 22 were unilateral lung, 10 were bilateral lungs, and three had extrapulmonary relapses (extremity, cardiac, and paraspinal/rib).

**TABLE 1:** Patient demographics (*n* = 61 patients).

Variable	Surgical approach to initial pulmonary metastases				
	Thoracotomy (*n* = 9)	Thoracoscopy (*n* = 6)	CTT (*n* = 6)	Other/no surg/non‐met at dx (*n* = 40)	Total (*n* = 61)
Gender					
Male	5 (56%)	5 (83%)	2 (33%)	23 (57%)	35 (57%)
Female	4 (44%)	1 (17%)	4 (67%)	17 (43%)	26 (43%)
Median age at diagnosis (years)	13 (9, 18)	13.5 (7, 16)	13.5 (8, 17)	13.5 (5, 21)	13 (5, 21)
Ethnicity					
White/Non‐Hispanic	2 (22%)	1 (17%)	1 (17%)	10 (25%)	14 (23%)
Asian	0 (0%)	0 (0%)	0 (0%)	2 (5%)	2 (3%)
African American	2 (22%)	0 (0%)	0 (0%)	2 (5%)	4 (7%)
Hispanic	3 (34%)	4 (66%)	4 (66%)	20 (50%)	31 (51%)
Other	2 (22%)	1 (17%)	1 (17%)	5 (13%)	9 (15%)
Unknown	0 (0%)	0 (0%)	0 (0%)	1 (2%)	1 (2%)
Primary tumor site location					
Pelvis	0 (0%)	0 (0%)	1 (17%)	3 (8%)	4 (6%)
Extremity	9 (100%)	6 (100%)	5 (83%)	36 (90%)	56 (92%)
Trunk	0 (0%)	0 (0%)	0 (0%)	1 (2%)	1 (2%)
Primary extremity location					
Proximal femur	1 (11%)	0 (0%)	1 (20%)	1 (3%)	3 (6%)
Distal femur	3 (33%)	4 (67%)	1 (20%)	14 (39%)	22 (39%)
Proximal tibia	0 (0%)	2 (33%)	3 (60%)	12 (33%)	17 (30%)
Distal tibia	0 (0%)	0 (0%)	0 (0%)	1 (3%)	1 (2%)
Proximal humerus	4 (45%)	0 (0%)	0 (0%)	6 (17%)	10 (18%)
Proximal fibula	1 (11%)	0 (0%)	0 (0%)	2 (5%)	3 (5%)
Histological response of primary tumor					
Good responder (<10% viable tumor)	3 (33%)	2 (33%)	4 (66%)	12 (30%)	21 (34%)
Poor responder (≥10% viable tumor)	6 (67%)	3 (50%)	1 (17%)	23 (57%)	33 (54%)
Unknown	0 (0%)	1 (17%)	1 (17%)	5 (13%)	7 (12%)
Metastatic site at diagnosis					
Lungs only	7 (78%)	4 (67%)	6 (100%)	3 (8%)	20 (33%)
Lungs and bones	2 (22%)	2 (33%)	0 (0%)	0 (0%)	4 (6%)
None	0 (0%)	0 (0%)	0 (0%)	37 (92%)	37 (61%)
Laterality of pulmonary metastases at initial diagnosis					
Unilateral	2 (22%)	2 (33%)	3 (50%)	2 (67%)	9 (38%)
Bilateral	7 (78%)	4 (67%)	3 (50%)	1 (33%)	15 (62%)
Relapse (first) or death since diagnosis					
None	4 (44%)	1 (17%)	2 (33%)	3 (8%)	10 (16%)
Death	3 (34%)	1 (17%)	1 (17%)	10 (25%)	15 (25%)
Relapse	2 (22%)	4 (66%)	3 (50%)	27 (68%)	36 (59%)
Vital status at last follow‐up					
Alive	6 (67%)	5 (83%)	5 (83%)	30 (75%)	46 (75%)
Deceased	3 (34%)	1 (17%)	1 (17%)	10 (25%)	15 (25%)
Maximum # of relapse					
0	4 (44%)	1 (17%)	3 (50%)	3 (7%)	11 (18%)
1	5 (56%)	2 (33%)	0 (0%)	13 (33%)	20 (33%)
2	0 (0%)	2 (33%)	0 (0%)	12 (30%)	14 (23%)
3	0 (0%)	0 (0%)	1 (17%)	9 (23%)	10 (16%)
4	0 (0%)	0 (0%)	1 (17%)	3 (7%)	4 (7%)
5	0 (0%)	1 (17%)	1 (16%)	0 (0%)	2 (3%)

Abbreviation: CTT, combined thoracoscopy with thoracotomy.

### 3.2 Surgical outcomes

**FIGURE 1:**
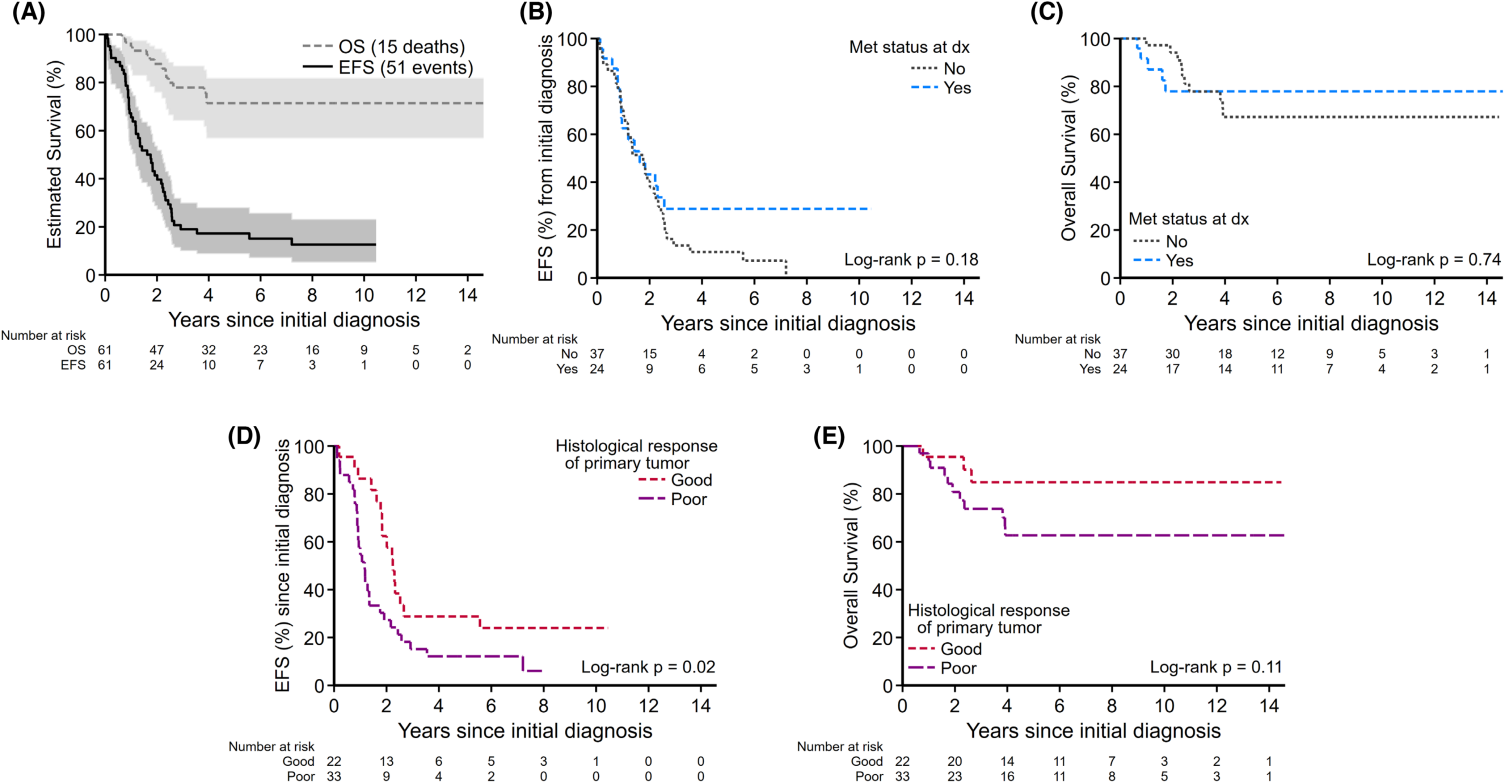
(A) EFS and OS of 61 patients with OST who underwent PM. (B) EFS for localized versus metastatic disease at diagnosis. (C) OS for localized and metastatic disease at diagnosis. (D) EFS for good histologic response and poor histologic response. (E) OS for good histologic response and poor histologic response. EFS, event‐free survival; OS, overall survival; OST, osteosarcoma; PM, pulmonary metastasectomy.

Of 61 patients with OST who underwent PM, the 5‐year EFS and OS were 17% (95% CI: 8.9%–27.9%) and 71% (95% CI: 56.9%–81.8%), respectively (Figure 1A). There was no statistically significant difference in survival outcomes between those with localized and those with metastatic disease at diagnosis (5‐year EFS localized vs. metastatic, 11% [95% CI: 3.4%–23.1%] vs. 29% [95% CI: 11.8%–47.9%]; *p* = 0.18 [Figure 1B]; 5‐year OS 67% [95% CI: 47.5%–80.9%] vs. 78% [95% CI: 54.7%–90.2%]; *p* = 0.74 [Figure 1C]). Patients with good primary histologic response had a significantly better EFS compared to those with poor primary histologic response (5‐year EFS good vs. poor histologic response, 30% [95% CI: 12.4%–50.4%] vs. 12% [95% CI: 3.8%–25.5%]; *p* = 0.02 [Figure 1D]; but histological response did not affect 5‐year OS; 84% [95% CI: 58.1%–94.6%] for good responders vs. 63% [95% CI: 42.6%–77.5%] for poor responders; *p* = 0.11 [Figure 1E]).

**FIGURE 2:**
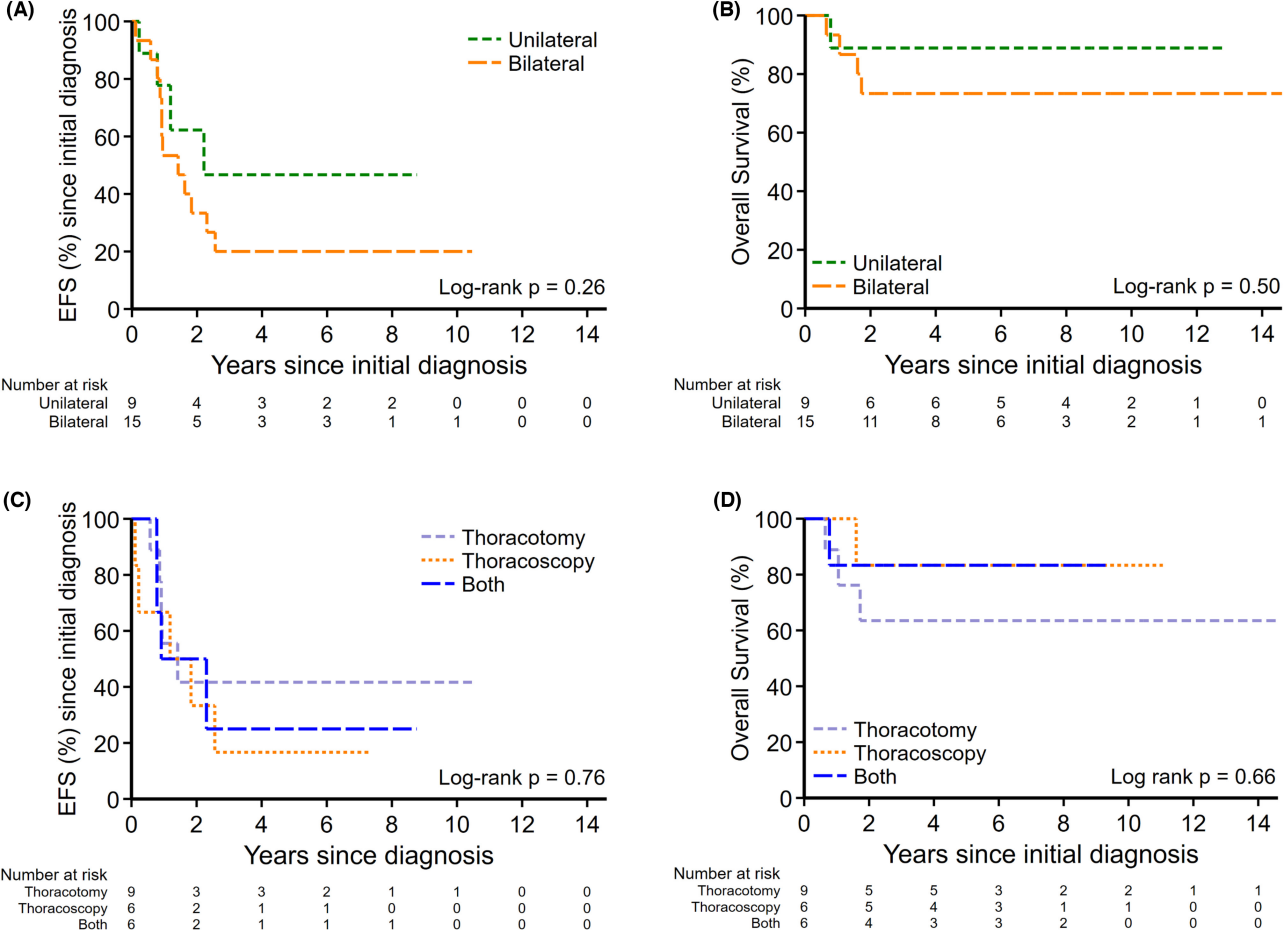
(A) EFS from initial diagnosis for patients with unilateral and bilateral pulmonary metastases at diagnosis. (B) OS for patients with unilateral and bilateral pulmonary metastases at diagnosis. (C). EFS from diagnosis with surgical approaches to initial pulmonary metastasectomy. (D). OS for surgical approaches to initial pulmonary metastases. EFS, event‐free survival; OS, overall survival.

Of 24 patients with metastatic OST at diagnosis, 21 patients underwent PM during primary treatment; nine (43%) had thoracotomy, six (28.5%) had thoracoscopy and six (28.5%) had CTT. The initial surgical approach to PM had no statistically significant bearing on survival outcomes (EFS *p* = 0.76, OS *p* = 0.66) from the time of initial diagnosis. Of the 50 patients that had at least one relapse, the surgical approach to their first relapse was not significantly different neither for EFS (*p* = 0.36) nor OS (*p* = 0.59). There was no significant difference in survival in patients with unilateral compared to those with bilateral pulmonary metastases (2‐year EFS: 62% [95% CI: 21.3%–86.4%] vs. 33% [95% CI: 21.1%–56.4%]; *p* = 0.26 [Figure 2A], and 2‐year OS: 89% [95% CI: 43.3%–98.4%] vs. 73% [95% CI: 43.6%–89.1%]; *p* = 0.50 [Figure 2B]). There was no difference in survival outcomes between surgical approach type to initial pulmonary metastases (2‐year EFS thoracotomy vs. thoracoscopy vs. CTT, 42% [95% CI: 10.9%–70.8%] vs. 33% [95% CI: 4.6%–67.6%] vs. 50% [95% CI: 11.1%–80.4%]; *p* = 0.76 [Figure 2C]; 2‐year OS 64% [95% CI: 23.8%–86.6%] vs. 83% [95% CI: 27.3%–97.5%] vs. 83% [95% CI: 27.3%–97.5%]; *p* = 0.66 [Figure 2D]).

**FIGURE 3:**
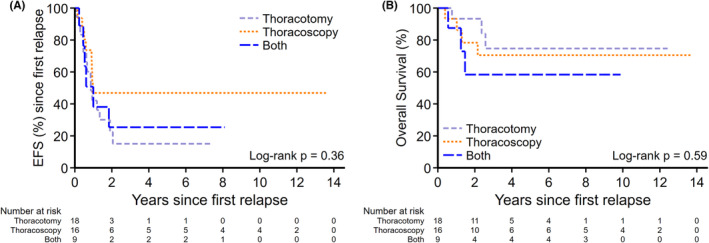
(A) EFS from first relapse for surgical approaches and first pulmonary relapse or progression. (B) OS for surgical approaches to first pulmonary relapse or progression. EFS, event‐free survival; OS, overall survival.

Forty‐three (*n* = 33 non‐metastatic at diagnosis; *n* = 10 metastatic at diagnosis including eight that underwent PM with primary therapy) had first pulmonary relapse or progression and underwent PM; 18 (42%) had thoracotomy, 16 (37%) had thoracoscopy, and nine (21%) had CTT with no significant difference in survival outcomes (2‐year EFS thoracotomy vs. thoracoscopy vs. CTT, 23% [95% CI: 6.3%–44.9%] vs. 47% [95% CI: 21.4%–68.9%] vs. 25% [95% CI: 3.8%–56.4%]; *p* = 0.36 [Figure 3A]; 2‐year OS 93% [95% CI: 61.3%–99%] vs. 78% [95% CI: 46.5%–92.5%] vs. and 58% [95% CI: 18%–84.4%]; *p* = 0.59 [Figure 3B]).

Nineteen of the 21 patients with pulmonary metastases at initial diagnosis who underwent PM had reported surgical morbidities (Table 2). A total of 14 thoracotomies, seven thoracoscopies and seven CTT were performed. The surgical approach to oligometastatic disease (<5 nodules) and multiple pulmonary nodules (five or more nodules) was evenly split between thoracotomy versus thoracoscopy in these patients (Table S1). There was no significant difference in the number of pneumothoraces in the thoracotomy group compared to the thoracoscopy or CTT groups (86% vs. 57%, 43%, respectively; *p* = 0.15). Requirements for epidural and/or PCA with or without continuous narcotic infusion were significantly less in the thoracoscopy group compared to the thoracotomy or CTT groups (29%, 79%, and 86% CTT, respectively; *p* = 0.048). There were significantly less chest tubes placed in the thoracoscopy group compared to the thoracotomy or CTT groups (43%, 86%, 100%, respectively; *p* = 0.03), but no difference in the duration of chest tube placement or hospitalizations (*p* = 0.46). None of the patients had postoperative infections. There was no difference in Foley catheter utilization (29% in thoracotomy, 0% in thoracoscopy, and 29% in CTT; *p* = 0.62).

**TABLE 2:** Post op morbidities initial resection (*n* = 21 patients/28 observations).

Variable	Surgical approach to initial pulmonary metastases				*p*‐Value
	Thoracotomy (*n* = 14)	Thoracoscopy (*n* = 7)	CTT (*n* = 7)	Total (*n* = 28)	
Post op infection					
No	14 (100%)	7 (100%)	7 (100%)	28 (100%)	NA
Post op pneumothorax/emphysema					
No	2 (14%)	3 (43%)	4 (57%)	9 (32%)	0.15
Yes	12 (86%)	4 (57%)	3 (43%)	19 (68%)	
Post op pain control medication					
No	3 (21%)	5 (71%)	1 (14%)	9 (32%)	**0.048**
Yes	11 (79%)	2 (29%)	6 (86%)	19 (68%)	
Post op Foley catheter					
No	10 (71%)	7 (100%)	5 (71%)	22 (79%)	0.36
Yes	4 (29%)	0 (0%)	2 (29%)	6 (21%)	
Median days of post op Foley catheter	3 (1, 5)	NA	2 (NA)	2 (1, 5)	0.62
Post op chest tube					
No	2 (14%)	4 (57%)	0 (0%)	6 (21%)	**0.03**
Yes	12 (86%)	3 (43%)	7 (100%)	22 (79%)	
Median days of post op chest tube	3 (2, 13)	5 (2, 5)	3 (1, 6)	3 (1, 13)	0.46
Post op hospitalization					
No	2 (14%)	2 (29%)	0 (0%)	4 (14%)	0.33
Yes	12 (86%)	5 (71%)	7 (100%)	24 (86%)	
Median days of post op hospitalization	4 (3, 14)	2 (1, 9)	4 (2, 12)	4 (1, 14)	0.48

*Note*: Two patients without any post operative morbidities listed above.

Abbreviation: CTT, combined thoracoscopy with thoracotomy.

Of the 43 patients that suffered a first pulmonary relapse/progression and underwent metastasectomy, 40 had morbidities recorded (Table 3). A total of 25 thoracotomies, 19 thoracoscopies and 8 CTT were performed. Majority of patients with multiple pulmonary nodules underwent thoracotomy (Table S2). There was no difference in postoperative infections between the three surgical groups (*p* = 0.15). There were fewer pneumothoraces in the thoracoscopy group compared to thoracotomy or CTT (53%, 72%, and 100% respectively; *p* = 0.05). There was a higher incidence of Foley catheter utilization in the thoracotomy group compared to the thoracoscopy or CTT groups (28%, 0%, and 13%, respectively; *p* = 0.03). There was significantly less epidural and/or PCA with and without continuous narcotic infusion use for the thoracoscopy group compared to thoracotomy or CTT (21%, 72%, and 88%, respectively; *p* < 0.001). Incidence of chest tube placement was comparable among all groups (*p* = 0.20). There were shorter hospitalization admissions in those that underwent thoracoscopy compared to thoracotomy or CTT (3, 5, and 6 days, respectively; *p* < 0.01), but no difference in the incidence of hospitalizations (*p* = 0.08).

**TABLE 3:** Post Op morbidities first relapse resection (*n* = 43 patients/52 observations).

Variable	Surgical approach to first relapse				*p*‐Value
	Thoracotomy (*n* = 25)	Thoracoscopy (*n* = 19)	CTT (*n* = 8)	Total (*n* = 52)	
Post op Infection					
No	25 (100%)	19 (100%)	7 (88%)	51 (98%)	0.15
Yes	0 (0%)	0 (0%)	1 (13%)	1 (2%)	
Post op pneumothorax/emphysema					
No	7 (28%)	9 (47%)	0 (0%)	16 (31%)	**0.05**
Yes	18 (72%)	10 (53%)	8 (100%)	36 (69%)	
Post op pain control medication					
No	7 (28%)	15 (79%)	1 (12%)	23 (44%)	**<0.001**
Yes	18 (72%)	4 (21%)	7 (88%)	29 (56%)	
Post op Foley catheter					
No	18 (72%)	19 (100%)	7 (88%)	44 (85%)	**0.03**
Yes	7 (28%)	0 (0%)	1 (12%)	8 (15%)	
Median days of post op Foley catheter	5 (3, 7)	NA	3 (NA)	4 (3, 7)	0.35
Post op chest tube					
No	8 (32%)	4 (21%)	0 (0%)	12 (23%)	0.20
Yes	17 (68%)	15 (79%)	8 (100%)	40 (77%)	
Median days of OST op chest tube	4 (2, 7)	2 (1, 10)	4 (3, 28)	3 (1, 28)	0.07
Post op hospitalization					
No	7 (28%)	1 (5%)	0 (0%)	8 (15%)	0.08
Yes	18 (72%)	18 (95%)	8 (100%)	44 (85%)	
Median days of post op hospitalization	5 (2, 8)	3 (1, 11)	6 (4, 29)	4 (1, 29)	**0.007**

*Note*: Three patients without any post operative morbidities listed above.

Abbreviations: CTT, combined thoracoscopy with thoracotomy; OST, osteosarcoma.

## 4 DISCUSSION

Aggressive PMs have been shown to improve survival in patients with metastatic OST since the 1970s.^11^ For decades, thoracotomy has been the primary surgical approach in order to manually identify radiographically undetectable lesions by palpating calcified microscopic OST nodules.^12^ Thoracotomy with manual exploration of lungs continues to be the recommended surgical approach in metastatic OST. In contrast, since the emergence of video‐assisted thoracoscopic surgery (VATS) in the early 1990s as a minimally invasive alternative approach for lung cancer surgery, VATS has been established as the gold standard for surgical resection of early‐stage lung cancer,^13^ and is increasingly being utilized to resect pulmonary metastases in many cancers, including OST. Despite current intensive multimodal therapies for OST, pulmonary metastatic disease remains difficult to prevent and treat. Surgical resection of pulmonary metastases remains the mainstay of treatment for cure.^14^, ^15^, ^16^, ^17^, ^18^, ^19^, ^20^, ^21^, ^22^, ^23^, ^24^, ^25^, ^26^, ^27^, ^28^, ^29^ Controversy continues to exist in OST regarding the optimal metastasectomy approach for OST lung metastasis.

The increased sensitivity of CT scans has allowed detection of pulmonary micronodules (<10 mm) that were not previously appreciated.^30^ Despite the improvement of CT scans, the detection rate of pulmonary nodules remains incomparable to that of direct lung palpation.^31^ CT scan has been reported to underestimate the number of metastatic lesions in OST.^32^ However, the prognostic significance of these micronodules has not been fully established. Davison et al. recently reported comparable 5‐year OS in patients with pulmonary nodules <5 mm at diagnosis compared to patients with no nodules, suggesting that surgery may not be necessary for micronodules identified at initial diagnosis.^33^ However, this cohort of patients were therapy‐naïve with micronodules that likely responded to standard primary systemic therapies compared to those with relapse or progressive pulmonary disease that persisted after multiple systemic therapies. In contrast, Ahmed et al performed a retrospective study in 125 patients with metastatic OST (48 with initial lung metastases at diagnosis and 77 with delayed lung metastases), and reported that patients with high ratio (>1) of surgically resected (SR) to radiologically detected (RD) OST pulmonary nodules (SR/RD) were associated with poor survival compared to patients with low ratio of SR/RD.^34^ They proposed that the poor survival among patients with high ratio of SR/RD may have had unresected subclinical lung disease, inferring that the presence of unresected micronodules conferred a poor prognosis.^34^

Studies assessing the impact of different PM approaches in OST are lacking. Importantly, our study reports comparable survival outcomes between thoracotomy versus thoracoscopy versus CTT at initial metastasectomy and at first pulmonary relapse or progression. Contrary to other published reports, there was no significant difference in OS with regards to primary tumor histologic response and in EFS/OS with regards to the laterality of pulmonary disease at diagnosis.^35^ However, this could be attributed to our small sample size. Those majority of patients with localized disease at initial diagnosis that relapsed, developed pulmonary metastases. This suggests the presence of neoadjuvant chemotherapy‐resistant micrometastases in our localized patient cohort. In contrast, our metastatic patient cohort likely had chemotherapy‐sensitive micrometastases at diagnosis, thus reflecting the lack of survival difference between localized and metastatic disease. Moreover, patients who had chemotherapy‐sensitive disease and received appropriate surgical management were able to achieve better OS indicating that prudent surgical management of metastatic sites contributes to better OS in OST patients that develop pulmonary metastases.

Our study suggests that the incidence of pneumothoraces is comparable among all groups at initial metastasectomy but significantly less for the thoracoscopy group at first relapse metastasectomy. Pain was significantly more in the thoracotomy and CTT groups at initial metastasectomy and at first relapse/progression, which could be due to repeated open thoracotomy incisions or extensive dissection caused by previous scarring leading to increased neuropathic pain. This is consistent with other reports with approximately 5%–80% of patients experiencing significant pain 2 months or more after thoracotomy, with approximately 30% of patients with persistent pain for 4–5 years after thoracotomy.^36^, ^37^

Several studies have reported the benefits of thoracoscopy in reducing surgical morbidity and postoperative morbidities such as fewer blood transfusions, earlier chest tube removal, and shorter hospital length of stay.^13^ We also noted less morbidity (chest tube, pain, Foley catheter and length of hospitalizations) in our thoracoscopy group. Objectively, thoracoscopy has been shown to better preserve shoulder function compared to thoracotomy, better respiratory function and less inflammatory activation and immune disruption.^38^, ^39^, ^40^, ^41^, ^42^, ^43^, ^44^ More importantly, reduced surgical morbidity also increases the likelihood of patients completing adjuvant chemotherapy and receiving it without any delay.^13^ Although pain is reportedly less in thoracoscopic approach, studies have shown that majority of patients experience chest wall paresthesia up to 19 months after thoracoscopy.^45^ Despite the benefits of thoracoscopy compared to thoracotomy, thoracotomy is the only method where direct lung palpation can be performed. Manual palpation remains a vital method to ensure all palpable metastatic pulmonary lesions are resected especially those not identified on CT scans, thereby maximizing patients' chances of survival against an inherent resistant disease. It is unclear whether the increasing uses of VATS for PM compared to thoracotomy decreases the confidence of the operating surgeon to palpate small pulmonary metastases.

Historically, patients who underwent PM have a 5‐year OS between 11% and 68%.^46^, ^47^, ^48^, ^49^ Our study reports a similar 5‐year OS. Most recently, Lautz et al. conducted a multi‐institutional retrospective review of patients with OST who underwent PM and reported no significant difference in 5‐year pulmonary disease free survival or OS between thoracotomy and thoracoscopy.^50^ Our study reports similar findings with additional significant secondary morbidities associated with thoracotomy and CTT such as increased duration of hospitalizations, pain, pneumothorax, incidence of chest tube placement and Foley catheter utilization. Limitations of this study include the single institution experience, small sample size, its retrospective design not allowing to assess competing risks, and bias to surgical approach based on standard of care and surgeon preference. Thoracoscopy, thoracotomy or CTT, each has its inherent risks and benefits. Surgical intervention for pulmonary metastases is necessary but not without risk or morbidity. Well‐designed prospective studies are required to fully elucidate the benefits of different surgical approaches.

## 5 CONCLUSIONS

Our patients with OST pulmonary metastases have comparable poor survival outcomes despite varying surgical approaches to PM. While our study reinforces the clinical equipoise between thoracotomy and thoracoscopy, we could not assess for surgical bias and other competing risks given the limitations of a retrospective study. This will be addressed in the ongoing Children's Oncology Group trial AOST2031 (NCT05235165), a prospective randomized clinical trial evaluating outcomes between thoracotomy and thoracoscopy in the management of pulmonary metastases for OST.

## AUTHOR CONTRIBUTIONS

**Christopher Kuo:** Conceptualization (lead); data curation (lead); formal analysis (lead); investigation (lead); methodology (lead); project administration (lead); visualization (lead); writing – original draft (lead); writing – review and editing (lead). **Jemily Malvar:** Conceptualization (supporting); data curation (supporting); formal analysis (lead); writing – review and editing (supporting). **Yueh‐Yun Chi:** Formal analysis (supporting); writing – review and editing (supporting). **Eugene S. Kim:** Writing – review and editing (supporting). **Rachana Shah:** Writing – review and editing (supporting). **Fariba Navid:** Writing – review and editing (supporting). **James Stein:** Investigation (supporting); writing – review and editing (supporting). **Leo Mascarenhas:** Conceptualization (lead); data curation (lead); formal analysis (supporting); investigation (supporting); methodology (lead); writing – original draft (supporting); writing – review and editing (lead).

## CONFLICT OF INTEREST STATEMENT

There are no conflict of interests.

## Supporting information


Table S1:

Table S2:

## Data Availability

Data can be made available upon reasonable request.
