# Supplementary material for: Survival outcomes and surgical morbidity based on surgical approach to pulmonary metastasectomy in pediatric, adolescent and young adult patients with osteosarcoma
Source: Cancer Med. 2023 Oct 6;12(20):20231–41. doi: 10.1002/cam4.6491 (PMC10652329; doi:10.1002/cam4.6491)
Supplement: Supplementary file 1 — Table S1: [file CAM4-12-20231-s002.docx]

**Supplementary table 1: Distribution of nodules at initial resection (n = 21 patients/28 observations)**

| Diagnosis number of nodules | Thoracotomy (n=14) | Thoracoscopy (n=7) | CTT (n=7) | Total (n=28) |
| --- | --- | --- | --- | --- |
| 5 or more nodules | 6 | 3 | 4 | 13 |
| Oligometastatic | 6 | 4 | 3 | 13 |
| No records | 2 | 0 | 0 | 2 |
