# Supplementary material for: Survival outcomes and surgical morbidity based on surgical approach to pulmonary metastasectomy in pediatric, adolescent and young adult patients with osteosarcoma
Source: Cancer Med. 2023 Oct 6;12(20):20231–41. doi: 10.1002/cam4.6491 (PMC10652329; doi:10.1002/cam4.6491)
Supplement: Supplementary file 2 — Table S2: [file CAM4-12-20231-s001.docx]

**Supplementary table 2: Distribution of nodules at first relapse resection (n = 43 patients/52 observations)**

| 1st Relapse number of nodules | Thoracotomy (n=25) | Thoracoscopy (n=19) | CTT (n=8) | Total (n=52) |
| --- | --- | --- | --- | --- |
| 5 or more nodules | 9 | 2 | 1 | 12 |
| Oligometastatic | 11 | 17 | 7 | 35 |
| No records | 5 | 0 | 0 | 5 |
